# A health promotion model-based intervention to enhance treatment adherence in patients with type 2 diabetes

**DOI:** 10.1186/s12889-024-19452-3

**Published:** 2024-07-19

**Authors:** Nahid Shahabi, Gholamali Javdan, Zahra Hosseini, Teamur Aghamolaei, Amin Ghanbarnejad, Ahmad Behzad

**Affiliations:** 1 Social Determinants in Health Promotion Research Center, Hormozgan Health Institute, Hormozgan University of Medical Sciences, Bandar Abbas, Iran; 2 Food Health Research Center, Hormozgan University of Medical Sciences, Bandar Abbas, Iran

**Keywords:** Treatment adherence, Type 2 diabetes mellitus, Education, Health promotion, Pender’s health promotion model

## Abstract

**Background:**

The present study aimed to determine the effect of an intervention based on Pender’s health promotion model (HPM) on treatment adherence in patients with type 2 diabetes (T2D).

**Methods:**

The present quasi-experimental study with a 3-month follow-up was conducted in Bandar Abbas, a city in the south of Iran in 2023. The intervention group (IG) with a total number of 95 T2D patients was selected from Hormuz diabetes clinic and the control group (CG) with 95 T2D patients was selected from comprehensive health centers through a clustering sampling method. The educational intervention was implemented in 10 sessions to improve patients’ treatment adherence. The teaching methods in training sessions were lectures, joint discussions, Q&A, role-play and peer training. The participants were evaluated using a researcher-made questionnaire including the constructs of Pender’s HPM about T2D treatment adherence, hemoglobin A1C (HbA1C), and BMI. Independent-samples t-test, paired-samples t-test, covariance analysis and stepwise regression analysis were used. Data analysis was done in SPSS 26.

**Findings:**

Three months after the intervention, in comparison to the CG, the mean and standard deviation of treatment adherence benefits (*p* = 0.002), treatment adherence self-efficacy (*p* = 0.010), treatment adherence related affect (*p* = 0.001), interpersonal influences (*p* = 0.012), commitment to plan of action (*p* < 0.001), treatment adherence behavior (*p* = 0.022), treatment adherence experiences (*p* = 0.001) was higher in the IG. The mean and standard deviation of situational influences (*p* < 0.001), immediate competing demands and preferences (*p* = 0.018) were lower than the CG. The results obtained from the analysis of covariance proved the effectiveness of the intervention in the constructs of Pender’s HPM and HbA1C in participants of the IG (*p* < 0.001). The regression analysis showed, after the intervention, for every 1 unit of change in commitment to behavior planning, action related affect and perceived self-efficacy, compared to before the intervention, there were 0.22 units, 0.16 units and 0.26 units of change in the behavior score in the IG.

**Conclusion:**

The findings proved the effectiveness of the educational intervention in improving the constructs in Pender’s HPM and the blood sugar level of T2D patients. As the results of the educational intervention showed, the use of a suitable educational approach as well as the development of appropriate educational content for the target population can significantly improve the treatment adherence behavior.

**Trial registration:**

This study is registered on the Iranian Registry of Clinical Trials (IRCT20211228053558N1: https://www.irct.ir/trial/61741) and first release date of 17th March 2022.

## Background

Among the most common chronic diseases in the world, diabetes is a continuous global threat to human health and global medical care [1]. As reported by the International Diabetes Federation, 10.5% of the adult population (20–79 years) on a global scale suffer from diabetes, and about half of them are not aware of living with this condition [2]. It is estimated that the Middle East and North Africa (MENA) regions including Iran will face the highest prevalence of diabetes in 2045, as the prevalence rate is predicted to reach a possible rate of 19.3% [2]. The prevalence of diabetes in Iran since the first national survey published in 1999 and despite the efforts and strategies to reduce the disease burden, has increased steadily and has become a national public health concern [3]. Diabetes is one of the 10 main causes of mortality in the world and Iran [4, 5]. The prevalence of diabetes and the total cases of diabetes in adults in Iran are estimated at 9.5% and 5,450,300 individuals, respectively [6].

T2D, which accounts for approximately 90% of all cases of diabetes, is associated with a variety of modifiable risk factors (e.g., unhealthy diet, obesity, physical inactivity, smoking and alcohol consumption) and non-modifiable factors (e.g., age, genetic factors and demographic factors) [7–9]. T2D patients are faced with a chronic disease that can lead to many complications and mortality [10]. The regular use of medicine, adopting a healthy lifestyle such as healthy eating, physical activity and psychosocial care are important in controlling T2D [11].

Adherence to treatment in diabetes is an important factor in achieving good diabetes control and preventing mortality [12]; nevertheless, evidence shows that treatment adherence is inadequate among these patients [13, 14]. Treatment adherence in patients with T2D has been estimated at 68% in the United States of America [15], 34% in India [16], 50% in Japan [17] and 29% in Ethiopia [18]. Iranian researchers have also drawn attention to the low adherence to diabetes treatment and found this rate to be 17% [19], 31% [20] and 40% [21] in a body of research.

Successful management of diabetes depends not only on drug therapy, but also on self-management such as self-care measures, balanced diet, physical activity, weight control, and self-monitoring of blood glucose [22]. Therefore, training and empowering patients is of a great importance [23]. Since treatment adherence is a complex behavior, health behavior theories and models can help researchers develop intervention programs [24]. Pender’s health promotion model (HPM) is a model to explain healthy behavior with a focus on the role of experience in shaping behavior. This model enables health experts to persuade people to adopt health-promoting behaviors using a psychosocial process [25]. This model, which is used as a mediational model in the present study, provides a framework for a deeper understanding of factors that lead to poor treatment adherence [26, 27]. Through examining these factors within the framework of Pender’s model, researchers can identify specific areas such as perceived barriers, increasing self-efficacy, and promoting perceived benefits to enrich interventions to improve treatment adherence [26, 27].

**Fig. 1:**
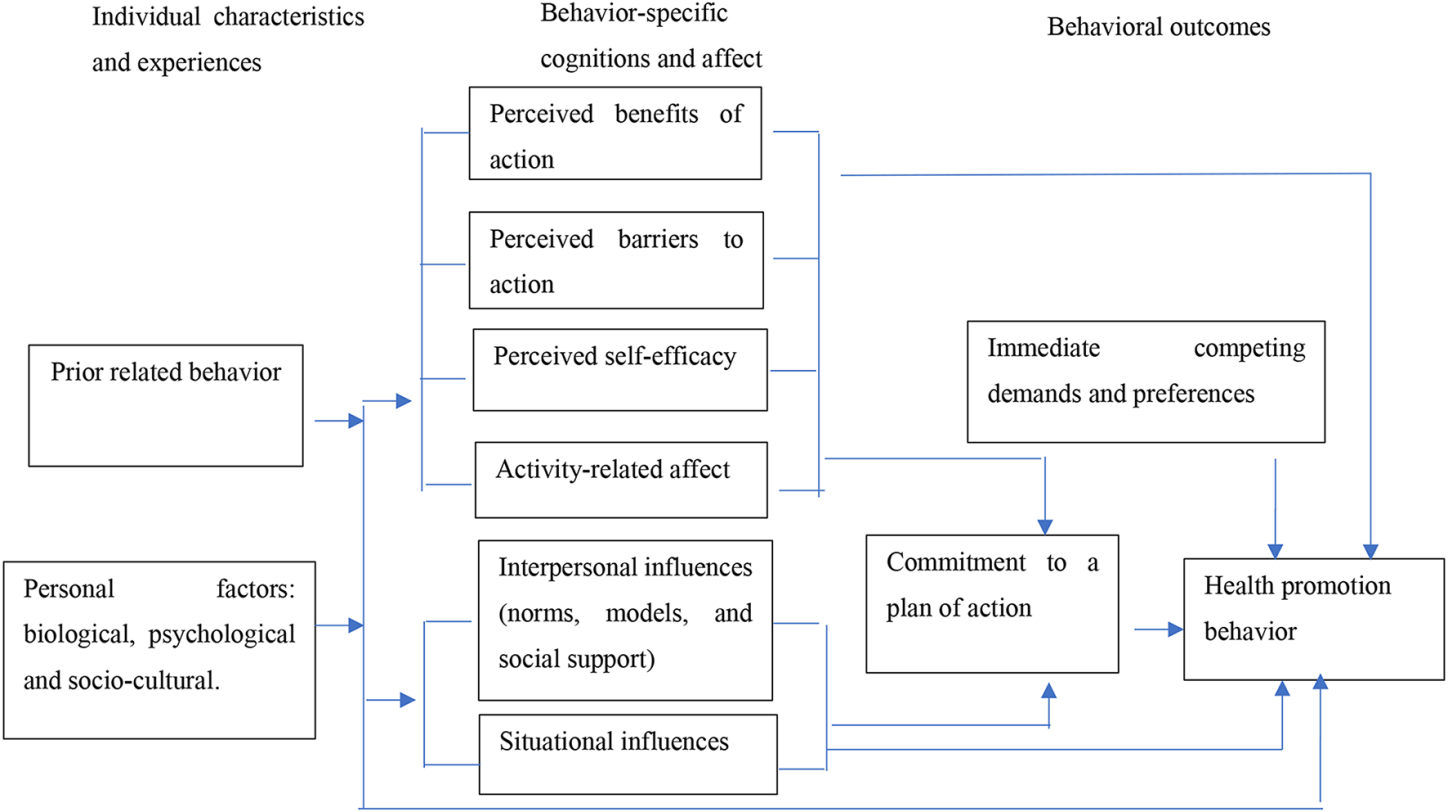
Pender’s health promotion model (HPM)

HPM includes three categories of factors, individual characteristics and experiences, behavior specific cognition and affect, behavioral outcomes (Fig. 1). Behavior-specific cognition and affect has modifiable constructs, including the perceived benefits of action, perceived barriers to action, perceived self-efficacy, activity related affect, interpersonal influences, and situational influences, that can lead to the adoption of (or a lack thereof) health promotion behaviors and resistance to immediate competing demands and preferences.

The prevalence of T2D is related to people’s lifestyle [28]. Unhealthy lifestyle marked by inactivity and unhealthy diet is common in southern Iran [29], which can affect the high prevalence of diabetes in this region [30] and T2D patients’ treatment adherence. There has been a dearth of research based on theoretical frameworks to promote adherence to type 2 diabetes treatment [31, 32]. Educational interventions based on Pender’s health promotion model have been carried out to adhere to treatment for other diseases [26, 33, 34]. Interventions based on this model in patients with T2D usually do not consider the set of treatment adherence behaviors together and only focus on one of these behaviors of regular medication consumption, physical activity, or diet [35, 36]. In this study, Pender’s HPM was used because the model constructs embrace personal, cognitive, affective and situational factors, all affecting the performance of healthy behaviors, especially adherence to T2D treatment [26, 34, 37]. Therefore, the present study aimed to design and implement an intervention based on Pender’s HPM to improve treatment adherence (medication, diet and physical activity) of T2D patients.

## Materials and methods

### Design and participants

The present quasi-experimental intervention was conducted with an intervention group (IG) and a control group (CG) on T2D patients in Bandar Abbas in January-March, 2023. This research had a pre-test, post-test design with a three-month follow-up, and aimed to improve treatment adherence in T2D patients using Pender’s HPM.

The research population consisted of T2D patients in Bandar Abbas. The intervention group was selected from Hormuz Diabetes Clinic, and the control group was selected from comprehensive health centers in the same city. The intervention was set in Hormuz Diabetes Clinic of Shahid Mohammadi Hospital, the greatest general hospital in Hormozgan province located in the south of Iran. The clinic is known as the largest diabetes clinic of the province.

### Inclusion and exclusion criteria

Inclusion criteria: willingness to participate in research, having a T2D medical record in Hormuz Diabetes Clinic (for IG) and comprehensive health centers (for CG), living in Bandar Abbas city, completion of an informed consent form to participate in the study.

Exclusion criteria: failure to participate in training sessions regularly (absence of more than 2 sessions), failure to visit the Hormoz diabetes clinic, absence in the post-test.

### Sample size and intervention sampling method

According to the existing literature [38], the combined standard deviation was estimated at 29.7, with an error of 5%, the test power of 80%. The difference between the treatment adherence score of the IG and CG was estimated at 13.5, and the sample size was estimated at be 76.

$$\:n=\frac{2{\left({z}_{1-\frac{a}{2}}^{2}+{z}_{1-\beta\:}\right)}^{2}{s}_{p}^{2}}{{({\mu\:}_{1}-{\mu\:}_{2})}^{2}}=76$$

To avoid the potential attrition, 25% was added to the above sample size and the final sample size was estimated at 95 in each of the two research groups. One participant from the IG did not complete the questionnaire.

**Fig. 2:**
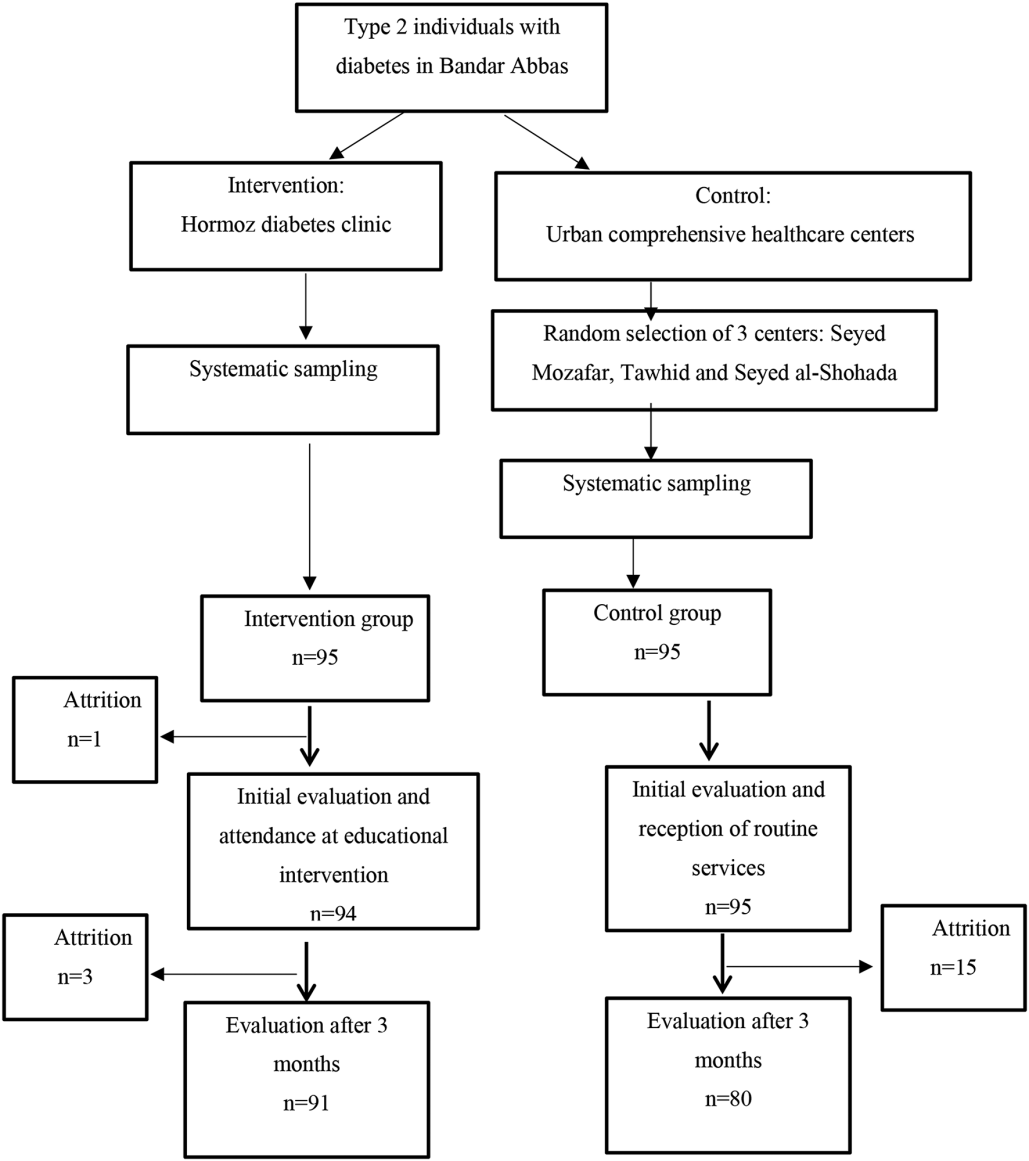
Sampling flowchart

The sampling was clustering in type. Hormuz diabetes clinic was considered as the intervention cluster and three comprehensive urban health centers as the control cluster (32 patients from Seyed Mozaffar clinic, 31 from Tawhid clinic and 32 from Seyed al-Shohda clinic). The latter was who not by any means related to the IG. For sampling in the IG, patients who met the inclusion criteria were included in the study through a systematic sampling based on the recorded case number. The same process followed for the CG using a list of individuals with diabetes recorded in the comprehensive health center registration system. In three months after the intervention, 91 patients in the IG and 80 in the CG completed the questionnaires (Fig. 2).

### Instrumentation

The measurement instruments used in this study were:

1. Questionnaire: Demographic variables and the constructs of Pender’s HPM, treatment adherence experiences and behavior-related questions.
2. Hemoglobin A1C (HbA1C).
3. BMI.

### Questionnaire

The questionnaire was developed in a qualitative study [39].

The first part of the questionnaire enquired about demographic variables, including age, sex, marital status, education level, employment status, economic status and history of diabetes, medication type and smoking.

The second part of the questionnaire enquired about the constructs of Pender’s HPM, treatment adherence experiences (derived from the qualitative study) and behavior related questions. It includes a total number of 65 questions, to be rated on a five-point Likert scale ranging from “strongly agree” to “strongly disagree” (strongly agree = 5, agree = 4, no idea = 3, disagree = 2, strongly disagree = 1), as follows:

Constructs of Pender’s HPM: “Perceived benefits of action” was rated along with 6 questions with a range of scores of 6 and 30. “Perceived barriers to action” was rated along with 7 questions that were reversely scored in a range of 7 to 35. “Perceived self-efficacy” included 8 questions, with a range of scores of 8 to 40. “Activity related affects” contained 7 questions, with a range of scores of 7 to 35. The last three questions of this construct were reversely scored to examine negative affect. “Interpersonal influences” was rated along with 7 questions, with a score range of 7 to 35. “Situational influences” consisted of 5 questions that were reversely scored with a score range of 5 to 25. “Immediate competing demands and preferences” was rated along with 7 questions that were reversely scored with a score range of 7 to 35. “Commitment to a plan of action” was rated along with 8 questions, with a score range of 8 to 40.

Treatment adherence experiences: “Treatment adherence experiences” included both personal experiences and others’ experiences with 5 questions, with a score range of 5 to 25.

Behavior: “Treatment adherence behavior” was measured along with 5 questions, with a range of scores of 5 to 25.

Quantitative content validity was checked using content validity ratio (CVR) and content validity index (CVI). The questionnaire was provided to 10 health education and health promotion specialists, internal medicine specialists, and Endocrinology & Metabolism experts. Moreover, to test the reliability of this instrument, test-retest and Cronbach’s alpha were used for each dimension of the questionnaire and the whole questionnaire. The test and re-test were conducted in the presence of 22 T2D patients visiting medical centers in Bandar Abbas to treat the disease. Two weeks after completing the first phase of the questionnaire, the patients were asked to answer the questions again. Cronbach’s alpha coefficient of the whole questionnaire was estimated at 0.924, which shows the acceptable reliability of the questionnaire. The questionnaire was distributed before and 3 months after the intervention.

### HBA1C

In the present study, blood sugar management was evaluated using the HBA1C test, known as a measure of the perceived benefits of glucose reduction in trials [40]. HbA1c was measured using whole blood samples via an enzymatic method using Biorex kits on an automatic biochemistry analyzer (Mindray BS-800). The test was administered before the intervention and three months after the intervention ended.

### BMI

Participants’ weight was measured to estimate BMI before and three months after the intervention. Weight was measured while subjects were minimally clothed without shoes using a digital scale with a sensitivity of 100 g, and height was measured in a standing position, without shoes, using a non-expandable tape measure with an accuracy of 0.5 cm.

### Data Collection

**Fig. 3:**
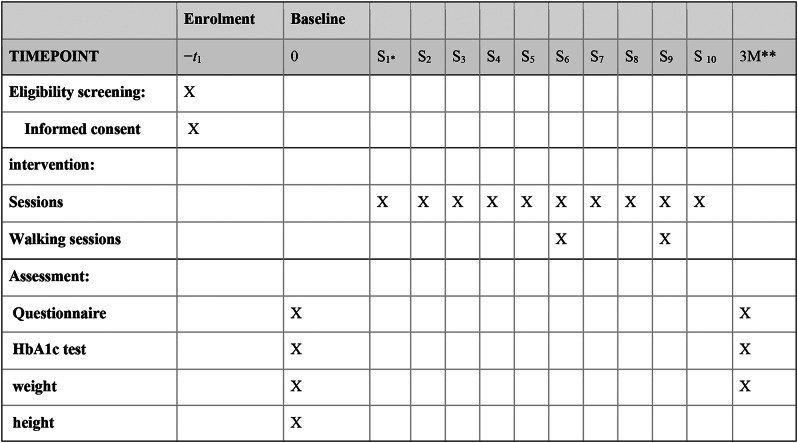
Timeline of intervention sessions and assessments. S* Session once a week, M** Month

The data were collected using a researcher-made questionnaire, HbA1C and BMI before and three months after the intervention from both the CG and IG (Fig. 3).

Before collecting the data, the necessary explanations about the objectives and procedure of study were provided to those who signed the consent form to participate in this research. Questionnaires were completed online in PorsLine. The researcher’s contact number was included in the first page. For those unable to complete the questionnaire for not having a smartphone, poor eye sight, or illiteracy, the content of the questionnaire was read out loud by the researcher and completed as they suggested. The researcher tried to reduce the drop-out rate by continuous follow-ups through phone calls, messages and attendance when people visit the clinic; however, a number of people were excluded from the study at different phases of study.

### Development of educational intervention

The educational intervention was made at two levels, individual and interpersonal. At the individual level, the intervention was made on T2D patients. At first, the patients were divided into four groups. Group-based training sessions were held in the afternoon hours in a room on the ground floor of Hormoz Clinic, which had adequate light and silence. The classroom was arranged in a U shape [41, 42]. The researcher stood in front of the audience as a speaker. This arrangement, which is mostly used for press conferences, training classes and collaboration meetings or brainstorming workshops, encourages people to participate and interact with each other. Therefore, the classes proceeded in a completely cooperative and friendly manner.

The intervention team consisted of a Ph.D. student of health education and health promotion, two experts in health education and health promotion, an internist and a master of nutrition. The team developed the content of intervention based on the data obtained from the pre-test and using the latest available resources, especially the American Diabetes Association. Two patients (1 male and 1 female) among T2D patients, found with a good treatment adherence, were present in the training sessions as role models. These role models viewed their presence and sharing of lived experiences was motivating and promising for treatment adherence.

The educational intervention was held for 2 months from January 21, 2022 to March 19, 2022. The sessions were held on a weekly basis, and the researcher allocated one day a week to patients who failed to attend the previous training session in their group for any reason, and held a make-up training session for them. The sessions were held as collaborative group discussion and Q&A. The self-efficacy related sessions on, for example, insulin consumption or blood sugar measurement, a blood sugar test device was used along with role play. It included a follow-up of treatment adherence behavior as a daily self-assessment program including date, time and content of breakfast, lunch and dinner, time and content of exercise, fasting sugar level and foot care instructed the next session. At the beginning of each session, the researcher and the participants discussed the previous session for approximately 5 min, and participants’ comments and criticisms were used to improve other sessions. In the final session, each participant was given two stickers “I adhere to diabetes treatment!” and “I am a healthy individual with diabetes.“. The participants were asked to stick the corresponding stickers to their home kitchen or to their work station. The educational media used during the intervention consisted of educational pamphlets, insulin poster (A4 size) and educational video (on insulin consumption and hypoglycemia). The duration of sessions was approximately 60–90 min; their length depended on the topic and participants’ reception. Some sessions took up to 120 min. Finally, 10 sessions were developed for intervention including eight face-to-face sessions and two online sessions (Table 1).

**Table 1:** Content of intervention sessions

Session no	Time	Topic	Mentioned Items
1	60 min	treatment adherence related affect + Commitment to plan of action + treatment adherence experiences	A leaflet containing the daily self-assessment program was delivered to the participants. The process of diabetes and the need to adhere to diabetes treatment were explained. Strategies to overcome negative affects were suggested and participants talked about their (positive and negative) emotions while showing treatment adherence behavior (medication, nutrition and physical activity). An online yoga training program was sent to the participants.
2	90 min	Commitment to plan of action + treatment adherence barriers	This session was held in the presence of companions. Information was provided about the necessity of planning to adhere to the treatment and commitment to the program. Participants’ experiences of planning and their level of adherence were discussed.
			Different barriers that may arise to treatment adherence and solutions to overcome them were discussed and then, they cooperated to come up with solutions to overcome these barriers. Free smartphone apps were introduced to plan treatment adherence activities. Strategies to overcome drug forgetfulness were discussed.
3	60 min	Commitment to plan of action + treatment adherence self-efficacy + treatment adherence experiences	The importance and manner of physical activity were discussed according to people’s individual and situational conditions. Two patients with diabetes who had been doing physical activity continuously for more than 10 years with different conditions and existing barriers were invited as role models to share experiences. They talked about the positive experiences of controlling and checking blood sugar, the necessity of ensuring blood sugar status and carrying the right amount of medicine for different life plans such as travel.
4	100 min	treatment adherence self-efficacy + treatment adherence benefits	The various benefits of each treatment adherence behavior were discussed. Then, two insulin users were asked to do the injection step by step, and emphasize the timeliness of the process and the precision. The participants were asked to check the process of blood glucose testing with a home blood glucose meter. They were taught the importance of nutrition in diabetes and the diabetic food plate, and participants were asked to try this technique in at least one meal before the upcoming session.
5	120 min	treatment adherence self-efficacy + treatment adherence benefits + Immediate competing demands and preferences + treatment adherence experiences + situational influences	Participants talked about their last week experience of implementing the pre-packaged food plate. Some healthy recipes and healthy desserts were also recommended. Temptation against sugary substances was discussed, and the researcher suggested strategies to control and regulate appetite and meals.
			Experiences were shared about immediate competing preferences, such as preference for sleep and television programs on adherence behavior and the expected outcome.
			Concerning the diabetic foot ulcer, the different outcomes and different care strategies were discussed. The importance of wearing appropriate socks and shoes for diabetics was discussed, as well as foot care in different situations such as travel, sports, beach, etc.
6	60 min	treatment adherence benefits + treatment adherence related affect	Stress management and anger control in diabetes were discussed. So were the different experiences of mental state and how it related to blood sugar. Emphasis was put on the different benefits of treatment adherence behaviors such as appropriate weight, proper nutrition and physical activity, positive affect while walking, higher work efficiency, blood sugar control, reduced hospitalization rate and reduced diabetes outcomes.
7	90 min	treatment adherence barriers + interpersonal influences	The importance of using the capacity of social support in treatment adherence was discussed and solutions were offered for better doctor-patient communication and to positively affect treatment.
			The benefits and barriers were analyzed by the researcher. The economic barriers and issues of medical, medicinal and diagnostic costs, and solutions such as time management were taught to the participants.
8	120 min	situational influences + Immediate competing demands and preferences	This session was held entirely on situational influences as well as competing preferences and demands. This session was called “Living with diabetes in different conditions”. A scenario was presented for a hypothetical patient with diabetes, and the participants were asked to analyze and guide the patient’s treatment adherence in different conditions.
			The conditions of adherence to treatment in various conditions such as domestic and foreign travel, heat and cold, infectious diseases, formal and informal gatherings were discussed and the researcher labeled each of the participants as “I adhere to the treatment of diabetes!” and “I am a healthy diabetic”.
9	15 min- online	treatment adherence self-efficacy	The common complications of T2D were discussed. Mention was then made of physical activities such as not doing sports in which the head is placed lower than the body when there is retinal damage.
10	15 min- online	situational influences + Immediate competing demands and preferences	In this session, considering the specific cultural conditions of the host country during Nowruz and the holy Ramadan, the recommendations approved by the Ministry of Health for diabetic patients were explained in simple language.

The latter was held as an online group meeting with all participants present in maximum 15 min. In this group, useful short messages on treatment adherence were also included. Some participants did not have smartphones, were illiterate, or failed to attend online classes due to poor eye sight. Therefore, their companion, a family member, cooperated in solving this problem and his/her contact number was added to the group. Besides, two walking sessions were planned in March for those who participated in each session as they preferred. At the interpersonal level, the companions in treatment adherence were present in two sessions. They were also invited to participate in the walking sessions. The researcher’s contact number and information were provided to the participants so that they could contact him if they had any queries. If any of the participants suffered from T2D complications, they were sent to the relevant specialist at Hormuz specialized and sub-specialized clinic, and the researcher made an appointment for them in the clinic. The data collection was simultaneous for both CG and IG. After the study, to adhere to research ethics and to acknowledge the CG participants’ involvement, they were also given the educational intervention materials. The CG did not have any training until the intervention ended.

### Data analysis

The data analysis was done in SPSS 26, using descriptive statistics, mean, standard deviation, minimum and maximum scores for interval variables and frequency and percentage for non-interval variables. Number 7 was set as the cutoff point for HBA1C according to Wulandari’s study [43]. Independent-samples t-test, paired-samples t-test, covariance analysis and stepwise regression analysis were used to check the effectiveness of behavior change intervention and Pender’s health promotion model constructs.

### Ethical considerations

To conduct the present research, the required permissions were gained from the Research Ethics Committee of Hormozgan University of Medical Sciences. This research was approved by the Ethics Committee of Hormozgan University of Medical Sciences with the ethical code of 377 IR.HUMS.REC.1400. To ensure voluntary participation in the study, informed consent forms were signed by the participants. The participants were assured that participation in the study was completely voluntary and that they could withdraw from the study any time at any phase of data collection if they did not wish to cooperate. The participants were assured that they could be informed of the findings of study if they wished. After the research was done, the educational materials and content were provided to the CG.

## Results

The majority of participants were female (2.70% in the intervention, 68.4% in the control), married (2.86% in the intervention, 84.2% in the control) and held a diploma (28.7% in the intervention, 23.2% in the control). The mean and standard deviation of participants’ age was 10.15 ± 54.93 in the IG and 11.20 ± 52.55 in the CG. The history of diabetes was 5.92 ± 10.6 for the IG and 5.80 ± 9.95 for the IG. Other demographic features of research participants in each group are summarized in Table 2. No significant difference was found in all contextual variables investigated in the IG and CG.

**Table 2:** Comparison of research participants’ demographic information

Variable	Group	Intervention group *n* (%)	Control group *n* (%)	*p*-value
Gender	Female	66(70.2)	65 (68.4)	0.789
	Male	28(29.8)	30 (31.6)	
Educational level	Illiterate	13(13.8)	18 (18.9)	0.844
	Primary school	24(25.5)	23 (24.2)	
	Secondary school	11(11.7)	12 (12.6)	
	Diploma	27(28.7)	22 (23.2)	
	University	19(20.2)	20 (21.1)	
Marital status	Single	3(3.2)	10 (10.5)	0.062
	Married	81(86.2)	80 (84.2)	
	Divorced	0(0)	1 (1.1)	
	Widowed	10(10.6)	4 (4.2)	
Occupation Status	employee	16(17.1)	14 (14.7)	0.732
	retired	20(21.3)	21 (22.1)	
	Student	7(7.4)	9 (9.5)	
	Unemployed	44(46.8)	39 (41.1)	
	housewife	7(7.4)	12 (12.6)	
Living with	nobody	4(4.3)	7 (7.4)	0.709
	spouse	15(15.9)	13 (13.7)	
	children	69(73.4)	71 (74.7)	
	other	6 (6.4)	4 (4.2)	
SES	good	24(25.5)	24 (25.3)	0.182
	middle	56(59.6)	47 (49.5)	
	low	14(14.9)	24 (25.2)	
Smoking status	cigarette	11 (11.7)	11 (11.6)	0.781
	hookah	18(19.1)	13 (13.7)	
	drugs	4(4.3)	4 (4.2)	
	non	61(64.9)	67 (70.5)	
Medicine	insulin	24(28.7)	25 (26.3)	0.748
	oral	53(56.4)	52 (54.7)	
	Insulin + oral	14(9.14)	18 (18.9)	
Age, yrs. mean ± SD		54.93 ± 10.15	52.55 ± 11.20	0.128
Diabetes history, yrs. mean ± SD		10.6 ± 5.92	9.95 ± 5.80	0.448
Family members mean ± SD		4.09 ± 1.80	4.18 ± 1.92	0.730

The scores obtained in the IG and CG in the time span before the intervention and 3 months after the intervention are shown in Table 2. The between-group differences in the mean scores of all constructs except for perceived barriers were statistically significant after the intervention. Concerning Hba1c and BMI after the intervention, there was no significant difference between the IG and CG. Comparison of the mean difference between the IG and CG showed that after the intervention in the IG, there was a statistically significant difference in the mean scores of all constructs except the perceived affects, behavior and BMI. In the CG, after the intervention, the mean scores of situational influences and immediate preferences were significant. (Table 3)

**Table 3:** Comparison of mean scores of Pender’s constructs before intervention and 3 months after intervention in the control group and intervention group

Variables	Groups	Independent analysis mode between intervention and control group		Paired-Samples T Test for each group (intervention, control)	
		Before intervention (Mean ± SD)	3 months after intervention (Mean ± SD)	Differences (Mean ± SD)	*p* value
Treatment adherence benefits	Intervention	24.96 ± 4.09	26.76 ± 2.67	1.72 ± 3.36	< 0.001
	Control	24.56 ± 5.11	24.86 ± 4.95	0.10 ± 1.05	0.397
	*p-value*	0.554	0.002		
Treatment adherence barriers	Intervention	22.16 ± 6.49	20.74 ± 6.27	-1.53 ± 3.45	< 0.001
	Control	21.87 ± 6.46	22.52 ± 6.40	0.27 ± 1.87	0.192
	*p-value*	0.762	0.067		
Treatment adherence self-efficacy	Intervention	30.02 ± 5.24	32.05 ± 4.75	2.09 ± 2.47	< 0.001
	Control	30.19 ± 5.22	30.08 ± 5.13	-0.09 ± 1.06	0.462
	*p-value*	0.825	0.010		
Treatment adherence related affect	Intervention	26.48 ± 4.06	27.03 ± 3.77	0.50 ± 5.92	0.418
	Control	25.63 ± 4.75	25.08 ± 3.44	-0.79 ± 6.19	0.258
	*p-value*	0.189	0.001		
Interpersonal influences	Intervention	29.73 ± 4.52	30.84 ± 3.07	1.07 ± 3.20	0.002
	Control	29.08 ± 4.14	29.45 ± 4.01	0.17 ± 1.27	0.222
	*p-value*	0.304	0.012		
Situational influences	Intervention	16.13 ± 4.11	13.45 ± 4.59	-2.80 ± 4.12	< 0.001
	Control	15.51 ± 4.93	16.24 ± 4.81	0.34 ± 1.02	0.004
	*p-value*	0.347	< 0.001		
Immediate competing demands and preferences	Intervention	20.69 ± 5.89	18.57 ± 5.59	-2.14 ± 3.98	< 0.001
	Control	20.13 ± 6.26	20.76 ± 6.40	0.29 ± 1.15	0.028
	*p-value*	0.523	0.018		
Commitment to plan of action	Intervention	26.34 ± 5.57	32.60 ± 6.41	6.37 ± 7.27	< 0.001
	Control	26.08 ± 5.97	26.45 ± 5.91	0.20 ± 1.30	0.172
	*p-value*	0.761	< 0.001		
Treatment adherence behavior	Intervention	18.02 ± 3.61	19.63 ± 4.34	1.71 ± 3.36	< 0.001
	Control	18.27 ± 3.22	18.19 ± 3.39	-0.32 ± 1.86	0.122
	*p-value*	0.613	0.022		
Treatment adherence experiences	Intervention	19.89 ± 3.79	21.32 ± 3.34	1.48 ± 3.06	< 0.001
	Control	18.99 ± 4.21	19.45 ± 4.19	0.10 ± 2.73	0.744
	*p-value*	0.123	0.001		
Hba1c	Intervention	9.14 ± 1.95	8.81 ± 2.03	-0.39 ± 0.64	< 0.001
	Control	8.95 ± 1.90	8.95 ± 1.93	0.18 ± 0.82	0.050
	*p-value*	0.494	0.626		
BMI (kg/m2)	Intervention	22.32 ± 1.90	21.75 ± 4.45	-0.57 ± 3.92	0.165
	Control	22.08 ± 2.08	22.12 ± 2.13	-0.035 ± 0.37	0.406
	*p-value*	0.402	0.496		

**Fig. 4:**
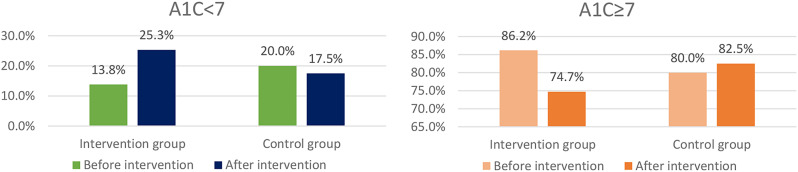
A1C changes in two groups before and after the intervention

According to Fig. 4, before the intervention, 13.8% of the IG had HbA1C less than 7. After the educational intervention, 25.3% of this group had HbA1C less than 7, and those with HbA1C greater than or equal to 7 decreased from 86.2 to 74.7% (Fig. 4).

To adjust for the effect of pre-test on the effectiveness of educational intervention on the post-test variables, covariance analysis was used. Between the adjusted mean scores of all constructs and also A1C, a statistically significant difference was observed between the IG and CG after the educational intervention. Therefore, the obtained results show the effect of intervention on variables of interest in participants of IG. Yet, concerning BMI, this difference was not statistically significant (Table 4).

**Table 4:** The results of covariance analysis of the effect of intervention on mean scores of Pender’s health promotion model constructs, behavioral experiences and A1C after intervention with pre-test effect controlled

Variables	Sum of squares	F	*P*-value
Treatment adherence benefits	124.06	27.83	< 0.001
Treatment adherence barriers	139.49	19.37	< 0.001
Treatment adherence self-efficacy	196.40	60.84	< 0.001
Treatment adherence related affects	174.31	13.45	< 0.001
Interpersonal influences	47.18	11.32	0.001
Situational influences	398.97	45.14	< 0.001
Immediate competing demands and preferences	244.91	28.89	< 0.001
Commitment to plan of action	1618.99	65.12	< 0.001
Treatment adherence behavior	100.01	14.28	< 0.001
Treatment adherence experiences	162.59	21.70	< 0.001
A1C	13.16	24.57	< 0.001
BMI	12.67	1.50	0.22

The results of multiple linear regression analysis using the difference in scores of behavior and constructs after intervention (in the posttest) compared to before the intervention (the pretest) in IG are summarized in Table 5. For each unit of change in commitment to plan of action in the posttest compared to the pretest, there was a change of 0.22 units in the behavior score of IG. Also, for each unit of change in treatment adherence related affect in the posttest compared to the pretest, there was a change of 0.16 units in the behavior score in the IG. Similarly, for each unit of change in perceived self-efficacy in the posttest compared to the pretest, 0.26 units of change occurred in the behavior score of IG.

**Table 5:** The results of step-by-step regression analysis of changes in the intervention group

Construct	B	Std. error	T	Standardized coefficients beta	*p*-value
Change in the scores of commitment to a plan of action	0.222	0.040	5.612	0.480	0.000
Change in scores of behavior-related affects	0.160	0.049	3.230	0.281	0.002
Change in scores of perceived self-efficacy	0.262	0.119	2.202	0.192	0.030

R Square = 0.367

## Discussion

The present study aimed to evaluate the effect of educational intervention based on Pender’s HPM on treatment adherence of T2D patients. As the results showed, after the intervention, there was a significant difference in treatment adherence behavior of the two groups. Moreover, the results of HbA1C were significantly different after the intervention in the IG. In the IG, there was a significant increase in perceived benefits, perceived self-efficacy, interpersonal influences, commitment to a plan of action, and treatment adherence experiences. There was a significant decrease in immediate competing demands and preferences and situational influences. There was a significant difference between the two groups. There was a significant difference between the two groups in the construct of treatment adherence related affects in the posttest. There was no significant difference in the IG before and after the intervention. There was no significant difference between the two groups in perceived barriers after the intervention, but there was a significant decrease in the IG before and after the intervention.

As the analysis showed, perceived benefits increased significantly in the IG, and there was also a significant difference between the IG and CG. The educational intervention managed to improve participants’ perceived benefits of treatment adherence behaviors. The higher the outcomes and benefits of treatment adherence, the more likely the willingness to perform the behavior increases. In several studies consistent with these findings, perceived benefits improved significantly after theory-based interventions such as Pender’s health promotion models, the health action process approach, and health belief [31, 32, 44–46]. Educating patients on benefits of physical activity, healthy diet and medications can facilitate the continuous performance of these behaviors. We suggest psychological-educational and cognitive-behavioral interventions be made for patients with a negative perception of the disease and low health literacy. The focus of interventions should be on individual’s perception of the disease and benefits of treatment adherence.

According to the present findings, the educational intervention managed to reduce participants’ perceived barriers scores in the IG. This result indicates that the participants were able to overcome the barriers after the educational intervention. These barriers include a wide range of physical, financial, access, and daily concerns that prevent us from treatment adherence behaviors. Similarly, the role of perceived barriers matters as non-adherence to treatment may ultimately delay the diagnosis of diabetes outcomes and have preventable consequences or premature death. Ranjbaran et al. contended that barriers to adherence to diet and medication intake were significantly reduced one month and six months after intervention in T2D patients [31]. Moreover, Shabibi et al. conducted an educational intervention based on the health belief model (HBM) and observed a significant change in the perceived barriers scores of the IG [32]. The high level of perceived barriers prevents one from adhering to treatment, puts health at risk and increases health care costs for both patients and society [47]. If the patient understands the essentiality of treatment, one’s motivation and willingness to remove barriers to self-care behaviors and adherence increase.

The perceived self-efficacy construct increased significantly in the IG, and there was a significant difference in the score of this construct between the IG and CG. For each unit of change in the perceived self-efficacy construct after the intervention compared to before the intervention, there was a change of 0.262 units in the behavior score of IG. It seems that the educational intervention in the present study based on Pender’s health promotion model and solutions such as role model, role play, guided exercises and goal setting managed to increase belief in one’s ability to correctly implement the behaviors of treatment adherence, healthy nutrition and physical activity. Ranjbaran et al. and Mohsenipouya et al. argued that self-efficacy significantly improved after the intervention [31, 46]. In Shabibi et al.‘s study, the educational intervention led to a change in the perceived self-efficacy scores of the IG [32]. Improving the self-efficacy of IG managed to facilitate their treatment adherence behavior. Perceived low efficacy to control a disease may lead to ineffectiveness in adopting healthy behavior or cognitive and emotional changes in representation of that disease. Higher control is associated with less anxiety, less avoidance of coping strategies and positive evaluation [48]. People adequate self-efficacy have good behavioral consistency to adhere to treatment.

The construct of interpersonal influences in the IG increased significantly, and there was also a significant difference in the score of interpersonal influences between the IG and CG. García-Pérez et al. maintained that in T2D patients, poor treatment adherence was significantly associated with a lack of family or social support [49]. In this study, we tried to encourage other companions to contribute more to patients’ treatment adherence by including family members in instructional sessions and sharing experiences of successful peers. A body of research suggested the effectiveness of educational interventions based on Pender’s HPM in interpersonal influences [36, 46, 50, 51]. However, Mohammadipour et al. could not significantly improve the scores of this construct [52], and these divergent findings can be due to different demographic characteristics of participants and interventions, because Mohammadipour’s research was conducted on T2D patients with a history of heart disease. Moreover, compared to the present study, it used a smaller sample size. Human communication plays an important role in adherence to chronic disease treatment. Clinicians, administrators, and policymakers should consider interpersonal interventions not only for their intrinsic value, but also for their potential to affect population health, patient experience, cost, and provider experience [53]. Lee et al. contended that patient-physician communication, especially information communication, had the potential to improve the patient therapeutic process through changes in medication adherence [54].

There was a significant decrease in the construct of situational influences in the IG, and there was a significant between-group difference in this score between the two groups. According to Pender’s health promotion model, each individual has a multidimensional whole that interacts with interpersonal and physical environments and plays an effective role in health promotion [46, 55, 56]. Therefore, it is important to pay attention to the role that the individual plays in family, workplace and society, as well as the physical environment and cultural characteristics of one’s place of residence in treatment adherence. In this study, the researcher tried to teach patients skills according to the above-mentioned characteristics so that they could better adhere to treatment. Other researchers also reported the effectiveness of intervention based on Pender’s HPM in situational influences [36, 51]. However, in the study of Goodarzi et al., this change of score was not statistically significant, which may be due to the participants’ pregnancy and their special conditions [57].

As the results showed, a significant increase was found in commitment to a plan of action in the IG in the posttest. There was also a significant difference between the IG and CG in terms of commitment to a plan of action. For one unit of change in commitment to plan of action after the intervention compared to before the intervention, there was a change of 0.222 units in the behavior score of the IG. Other researchers also reported the effectiveness of intervention based on Pender’s HPM in commitment to the plan of action [36, 46, 51, 57]. To increase this construct, it is not enough to emphasize this construct only; thus, other constructs of Pender’s model should also be taken into account. Conducting the educational intervention helped increase participants’ intention to set a goal to initiate and maintain adherence to drug therapy, physical activity, and nutrition. When health professionals seek to bring about behavioral changes in a group of people, targeting is usually included as part of their health promotion interventions [58, 59]. It seems that theory-based interventions, especially interventions based on Pender’s HPM, have been effective in improving commitment to a plan of action.

The results revealed a significant decrease in immediate competing demands and preferences in the IG. There was also a significant difference in the mean score of this construct between the two groups. Through increasing T2D patients’ ability of to manage daily life preferences and increasing control over unforeseen events, the educational intervention managed to help improve their decision-making in challenging situations. In line with the present findings, Rooh al-Amini et al. were able to have a significant effect on the score of IG in terms of immediate competing demands and preferences in their educational intervention [36]. In the study of Goodarzi et al., the IG had a significant decrease in the score of this construct after the educational intervention [57]. According to Pender’s HPM, competing demands may reduce commitment to a care plan, especially when the demands are urgent and overwhelming. However, if the health measures are attractive and accepted by the individual, commitment to treatment adherence behavior is reinforced [56]. Thus, while immediate competing demands and preferences can be important determinants of treatment adherence, there is limited evidence for the effectiveness of treatment adherence interventions to improve this construct.

In the present study, there was a significant increase in positive experiences of treatment adherence in the IG. There was also a significant difference between the IG and CG in the mean score of experiences. Arguably, these experiences gained by the individual and others have, on the one hand led to an increase in the individual’s self-efficacy, and on the other hand, to improve their perceived benefits because. For example, someone who has had a positive experience of regular medication consumption is now adequately aware of its benefits. More treatment adherence among patients with a longer history of the disease may result from their greater knowledge and experience of the disease, better doctor-patient relationships, and greater trust in physician recommendations [60]. Morse contended that by decreasing negative experiences and increasing positive experiences, treatment adherence is facilitated for children with tuberculosis as well as their caregivers [61]. Moreover, Taylor maintained that the regular collection of data on patients’ life experience can help make decisions about the social effects of health interventions [62]. As a result, interventions to improve treatment adherence can be effective in improving treatment experiences in various disease conditions such as T2D.

There was a significant difference between the two groups in terms of the behavior related affects after the intervention. The results of covariance analysis proved the effectiveness of intervention on this construct in the IG. For each unit of change in the behavior related affects in the posttest compared to the pretest, there was a change of 0.160 units in the behavior score of IG. The present study conducted an educational intervention to develop more positive feelings towards the behavior of treatment adherence and acceptance of disease conditions and overcome negative feelings such as the fear of testing, discomfort when avoiding food and taking medicine. It seems that the participants in IG managed to overcome their negative feelings and the positive feelings were strengthened. Other researchers also reported the effectiveness of intervention based on Pender’s HPM on the behavior related emotions [36, 51]. Also, Bağrıaçık reported that education provided based on Pender’s HPM helped individuals with diabetes develop a positive attitude towards insulin treatment [63]. Probably, using psychologists and counselors in interventions can be a suitable alternative to the behavior related affects.

The results of the present study showed that the IG had a lower HBA1C three months after the educational intervention ended. This finding shows that the framework designed according to Pender’s HPM for the intervention managed to positively affect the treatment adherence behavior and lead to the control of HBA1C. Hemoglobin A1C (HBA1C) is an important indicator of whether diabetes is well controlled and represents the average blood glucose level of the past 2 to 3 months. HBA1C is a good index for diagnosing diabetes, evaluating effectiveness, observing treatment adherence, and evaluating prognosis. It plays an important role in evaluating the occurrence and development of various complications of diabetes [64]. They reported an education based on Pender’s health promotion model [63]. Other studies also supported the present findings [65, 66]. Various interventions to improve treatment adherence have shown to be effective in improving HBA1C levels in patients with T2D, indicating the potential of these interventions to positively affect clinical outcomes.

In the present study, BMI of the IG decreased after the educational intervention, but this change was not statistically significant. It should be noted that the mean BMI score of IG was within the normal range before the intervention. Fouladvand et al. conducted an intervention with an emphasis on individuals with diabetes’ weight loss and reported the effectiveness of intervention in reducing the BMI of IG [67]. Mir et al. were able to achieve a significant weight loss after the educational intervention [68]. This difference in results can be attributed to the type of intervention and the participants’ characteristics, because the aforementioned interventions focused on weight loss and on overweight individuals with diabetes. However, in the present study, due to the participants’ normal BMI, the intervention did not focus on weight loss. In conclusion, while a body of research showed that BMI improved with systematic interventions, more research is needed to determine the most effective interventions to improve BMI in obese patients.

As the results showed, after the intervention, there was a significant difference between the two groups in terms of treatment adherence behavior. Farooghi maintained that the educational intervention based on Pender’s model was effective in adherence to treatment of patients with cardiovascular diseases [26]. Other studies also approved the effectiveness of intervention based on Pender’s HPM in treatment adherence behaviors of other diseases [33, 34]. The results reviewed in this study clearly show that educational programs, especially those based on Pender’s HPM, can significantly affect treatment adherence behaviors in chronic diseases such as diabetes. This model affects behavior at individual and interpersonal levels through using its useful constructs. Therefore, it is recommended to prepare and use such programs to improve these patients’ lives. Moreover, managers, planners and healthcare policymakers and other relevant authorities are advised to consider the implementation of detailed training programs based on Pender’s HPM to improve treatment adherence and other relevant factors and increase patients’ contribution to health promotion.

### Limitations

One limitation of the present study is sampling from one city and completing the questionnaire in the form of self-report. The current research was conducted only among people with diabetes living in Bandar Abbas city, and rural people were not included, which can be another limitation of the current research and reduce the generalizability of findings.

The strength of this study is that the researcher was fluent in the local language, and was able to communicate well with local patients, which could significantly simplify instructions for those in the IG yet not fluent in Persian. Using the hemoglobin HbA1C index along with the measurement of treatment adherence behavior is also one strength of the study.

## Conclusion

The present findings proved the effectiveness of educational intervention in improving the level of constructs in Pender’s HPM and the blood sugar level of T2D patients. As the results of the educational intervention showed, the use of a suitable educational approach as well as the development of educational content suitable for the target audience can significantly improve the treatment adherence behavior. It is suggested to arrange for healthcare centers to provide regular nutritional and psychological counseling for individuals with diabetes. The researchers suggest that future studies be conducted with a longer follow-up.

## Data Availability

The datasets generated during and/or analyzed during the current study are available from the corresponding author on reasonable request.

## Abbreviations

T2D: Type 2 diabetes mellitus
HPM: Pender’s health promotion model
HbA1C: Hemoglobin A1c test
BMI (kg/m2): Body mass index
CG: Control group
IG: Intervention group
